# PARP-1 Inhibition Rescues Short Lifespan in Hyperglycemic *C. Elegans* And Improves GLP-1 Secretion in Human Cells

**DOI:** 10.14336/AD.2017.0230

**Published:** 2018-02-01

**Authors:** Qianghua Xia, Sumei Lu, Julian Ostrovsky, Shana E McCormack, Marni J Falk, Struan F. A Grant

**Affiliations:** ^1^Division of Human Genetics, The Children’s Hospital of Philadelphia, Philadelphia, PA 19104, USA.; ^2^Division of Endocrinology and Diabetes, The Children’s Hospital of Philadelphia, Philadelphia, PA 19104, USA.; ^3^Department of Pediatrics, Perelman School of Medicine, University of Pennsylvania, Philadelphia, PA 19104, USA.; ^4^Institute of Diabetes, Obesity and Metabolism, Perelman School of Medicine, University of Pennsylvania, Philadelphia, PA 19104, USA

**Keywords:** *TCF7L2*, PARP-1, glucose, GLP-1, *C. elegans*

## Abstract

*TCF7L2* is located at one of the most strongly associated type 2 diabetes loci reported to date. We previously reported that the most abundant member of a specific protein complex to bind across the presumed causal variant at this locus, rs7903146, was poly [ADP-ribose] polymerase type 1 (PARP-1). We analyzed the impact of PARP-1 inhibition on *C. elegans* health in the setting of hyperglycemia and on glucose-stimulated GLP-1 secretion in human intestinal cells. Given that high glucose concentrations progressively shorten the lifespan of *C. elegans*, in part by impacting key well-conserved insulin-modulated signaling pathways, we investigated the effect of PARP-1 inhibition with Olaparib on the lifespan of *C. elegans* nematodes under varying hyperglycemic conditions. Subsequently, we investigated whether Olaparib treatment had any effect on glucose-stimulated GLP-1 secretion in the human NCI-H716 intestinal cell line, a model system for the investigation of enteroendocrine function. Treatment with 100uM Olaparib in nematodes exposed to high concentrations of glucose led to significant lifespan rescue. The beneficial lifespan effect of Olaparib appeared to require both *PARP-1* and *TCF7L2*, since treatment had no effect in hyperglycemic conditions in knock-out worm strains for either of these homologs. Further investigation using the NCI-H716 cells revealed that Olaparib significantly enhanced secretion of the incretin, GLP-1, plus the gene expression of *TCF7L2*, *GCG* and *PC1*. These data from studies in both *C. elegans* and a human cell line suggest that PARP-1 inhibition offers a novel therapeutic avenue to treat type 2 diabetes.

Type 2 diabetes (T2D) in many cases stems from an age-related deterioration in insulin signaling and glucose homeostasis. The pathogenesis of T2D is complex and incompletely understood. *Caenorhabditis elegans* (*C*. *elegans*) has been used as a model to study diabetes [1], because it has the evolutionary-conserved insulin-signaling pathway that is crucial to both aging and glucose metabolism, making it an informative system in which to study these processes [2].

In this study, we have used this model animal to investigate the functional significance of a finding derived from genome-wide association studies (GWAS), where many loci associated with T2D have been identified [3]. The signal within intron 3 of the *TCF7L2* gene on chromosome 10q25.2 represents one of the strongest genetic associations with T2D risk reported to date, and has been shown to be highly relevant across multiple populations [4, 5]. Furthermore, Bayesian modeling and studies leveraging the haplotypic diversity seen in populations of African ancestry have implicated rs7903146 as the causal SNP at this locus [6].

*TCF7L2* (formerly *TCF4*) is a well-established colorectal cancer gene, encoding a transcription factor that functions as the major effector of the canonical Wnt signaling pathway that plays multiple roles in cell fate determination, survival, proliferation and migration [7]. However, the ways in which *TCF7L2* may exert its influence on T2D risk has not yet been mechanistically determined [8]. A key clue is that this transcription factor binds to the promoter region of the proglucagon (*GCG*) gene [9]. Proglucagon is subject to post-translational processing by prohormone convertase 1 (PC1) to form glucagon-like peptide-1 (GLP-1), which functions as an incretin synthesized in intestinal L cells. As a gut hormone, GLP-1 secretion is regulated by the presence of nutrients in the lumen of the small intestine. GLP-1 has numerous physiological functions, including enhancement of glucose-stimulated insulin secretion, stimulation of β-cell anti-apoptosis and proliferation, and inhibition of glucagon secretion (resulting from different post-translational processing of proglucagon), food intake and gastric emptying [1]. The anti-diabetic properties of GLP-1 have generated intense interest in the use of this short peptide and its agonists for the treatment of patients with T2D [10, 11]. As such, one could postulate that TCF7L2 regulates GLP-1, representing one of a number of plausible mechanisms by which the *TCF7L2* locus modulates T2D risk.

In an effort to identify other putative mechanisms by which the *TCF7L2* locus impacts T2D, we previously used oligonucleotide pull-down followed by mass spectrometry analysis, to pinpoint specific protein complexes that bind across rs7903146. The most abundant member was poly [ADP-ribose] polymerase type 1 (PARP-1) [12]. Interestingly, a recent study also showed that the rheumatoid arthritis GWAS-implicated risk variant, CCR6DNP, regulates *CCR6* via PARP-1[13].

PARP-1 has been shown to play a role in chromatin structure [14]. Chromatin conformation has been shown to differ between alleles for rs7903146 in human pancreatic islets [15]. Interestingly, there have been other reports suggesting that the TCF7L2 and PARP-1 proteins interact [16], and indeed was confirmed in our previous study through the utilization of co-immunoprecipitation [12]. Also, *PARP-1* knockout mice have been shown to be protected from induced diabetes [16-18] [19]. Since PARP-1 inhibitors have already been used in humans for oncologic indications, compounds like Olaparib could be used potentially to reduce PARP-1 inhibitory effects on TCF7L2. We hypothesized that Olaparib-mediated PARP-1 inhibition would therefore result in activation of TCF7L2, and yield a net GLP-1 agonist effect.

To test this hypothesis on a whole-organism scale, we first investigated the effect of PARP-1 inhibition with Olaparib on the lifespan of *C. elegans* nematodes under varying hyperglycemic conditions. Subsequently, we investigated whether Olaparib treatment had any effect on glucose-stimulated GLP-1 secretion in a human NCI-H716 intestinal cell line, a model system for the investigation of enteroendocrine function. Collectively, these studies suggest that PARP-1 inhibition may offer a novel avenue to ameliorate the negative cellular and animal effects of hyperglycemia.

## MATERIALS AND METHODS

### C. elegans strains and maintenance

We compared the lifespan of *C. elegans* under hyperglycemic conditions (16 mM glucose or 25 mM glucose) in the presence or absence of Olaparib. The *C*. *elegans* strains used in the lifespan studies were wild-type N2 Bristol and *PARP1* knock-out mutant *pme-1*(ok988). Both strains were obtained from the *Caenorhabditis* Genetic Center (CGC, University of Minnesota). All nematodes were cultivated on nematode growth media (NGM) agar plates [20], which utilized living *Escherichia coli* bacteria OP50 as a food source, and were maintained at 20°C.

### RNAi experiment

*E. coli* HT115 (DE3) that contained the pop-1/pL4440 construct (obtained from Source BioScience) and empty RNAi feeding vector pL4440 (generously provided by Silencing Genomes) were used for NGM agar plates containing 1 mM isopropyl-β-D-thiogalacto-pyranoside (IPTG) and 25 ug/ml of carbenicillin. The actively growing *E. coli* were seeded drop- wise (100 ul) onto NGM/IPTG/carbenicillin plates the day before the experiments, covered with aluminum foil, allowed to dry and induced overnight at room temperature. Four conditions, with empty vector pL4440 or pL4440/pop-1 RNAi and with 0.1% DMSO (vehicle), or 100 uM Olaparib were performed with 16 mM glucose for each group consisting of n=100 age-synchronized worms.

### Lifespan Assay

In the lifespan assays, either water or 0.1% DMSO was used as vehicle. In the hyperglycemia group, a final concentration on NGM agar plates of either 16 mM glucose or 25 mM glucose was used, which corresponded to an estimated HbA1c plasma concentration of 11.7% or 17.3% in human diabetic patients. In drug treatment groups, a final concentration on NGM agar plates of 100 uM of the PARP-1 inhibitor, Olaparib (LC Laboratories, AZD-2281) was used with either 16 mM glucose or 25 mM glucose. To establish the concentration of the drug used in the study, a separate drug survival test was performed using N2 worms with Olaparib at the different concentrations of 0 uM, 10 uM and 100 uM. We further compared 10 uM and 100 uM of Olaparib in the high glucose condition (data not shown). For the majority of the lifespan runs, we did carry out biological repeats (Supplementary Fig. 1).

The experiments were performed on NGM agar on 35mm x 10mm Petri plates. Bacteria were seeded the day before each experiment. 150 ul solutions of each condition were added to the surface of NGM-agar plates inoculated with *Escherichia coli* bacteria OP50. 100 age-synchronized, day 0 (first day egg laying) young adult worms were spread evenly across five plates and exposed to each condition during the full adult life cycle. Worms were transferred to a new plate daily to prevent mixture of generations and depletion of food. Lifespan data were scored either daily or every other day. Worms that did not respond after repeated gentle stimulus were classified as dead. Worms that crawled away from the plate or harbored internally hatched worms were excluded from the study.

### Cell Culture

The human NCI-H716 cells were obtained from American Type Culture Collection (ATCC, Manassas, VA) and maintained in RPMI-1640 supplemented with 10% fetal bovine serum as described by ATCC.

### GLP-1 ELISA

NCI-H716 cells were grown in medium with or without 10 uM Olaparib. After 48 hours, supernatants were replaced by PBS containing 100 uM dipeptidyl peptidase IV inhibitor, diprotin A. Cells were subsequently stimulated for 30 minutes with or without 16 mM glucose. GLP-1 was measured by ELISA (EGLP-35K; Millipore, Billerica, MA) according to the manufacturer’s instructions.

**Figure 1.**
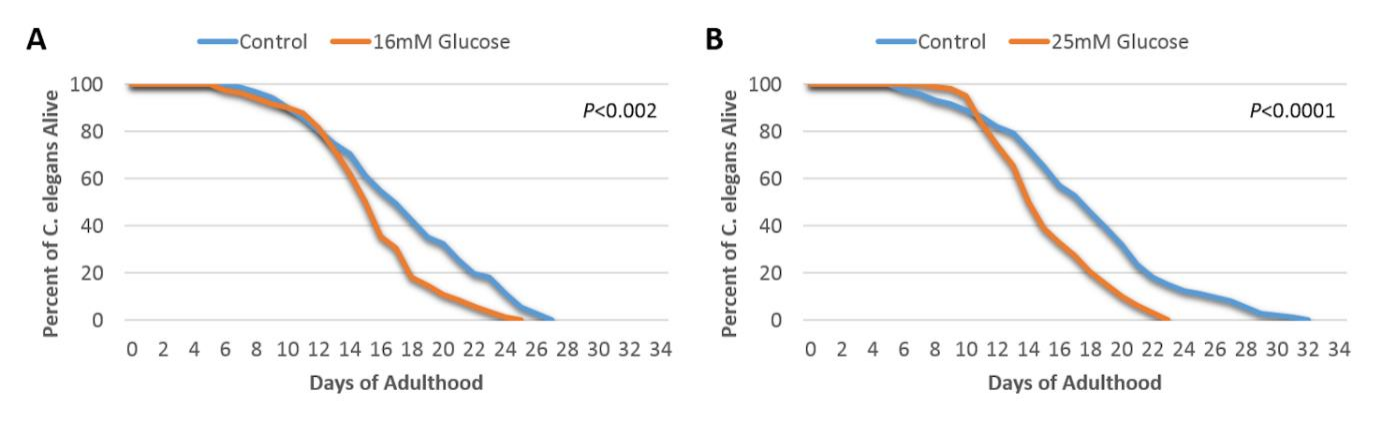
High glucose conditions shorten *C. elegans* lifespan Wild-type N2 *C. elegans* cultured under standard and high glucose conditions and subsequent lifespan assays. (**A**) Determination of lifespan for N2 worms unexposed or exposed to 16 mM glucose (*P*<0.002, log-rank test, n=100); (**B**) Determination of lifespan for N2 worms unexposed or exposed to 25 mM glucose (*P*<0.0001, log-rank test, n=100).

### RNA extraction, RT-PCR and real-time PCR

Total RNA was isolated from the cell pellets using Trizol Reagent (Invitrogen, Carlsbad, CA), according to the manufacturer’s protocol, and RNA concentrations were determined by spectrophotometer (NanoDrop ND-1000; Thermo Scientific, Wilmington, DE). Reverse transcription reactions were performed using High-Capacity RNA-to-cDNA™ Kit (Applied Sciences, Catalog Number: 4387406) according to the manufacturer’s protocol. Resultant cDNA was diluted 1:10 and used for quantitative real-time PCR reactions. The primer sequences used for amplification of *TCF7L2* were as follows: 5′-GCG GAA AGA CGG CCT CCG CCT CGC-3′ and 5′-GGC TTG TCT ACT CTG GAG GCT CCC-3′. The reactions were performed in a total volume of 15μl containing 2μl cDNA, 2x SYBR® Green Universal Master Mix (all real-time PCR reagents were obtained from Applied Biosystems, Foster City, CA). The reactions were run in a 384-well plate in ABI 7900 real-time PCR machine and analyzed with Sequence Detection Systems 7900HT version 2.4 software (Applied Biosystems, Foster City, CA). Briefly, samples were heated to 50° C for 2 minutes and then 95° C for 10 minutes to allow for DNA polymerase activation followed by 40 cycles of 95° C for 15 seconds and 60° C for 1 minute to allow for denaturing, annealing, and extension. Data were analyzed using the standard curve method with pooled cDNA serving as the standard. *TCF7L2* levels were normalized to GAPDH. The primers used for *GCG*, *PC1* and eukaryotic 18S rRNA were purchased from Life Technologies (Cat. # 4331182, 4331182 and 4453320). Proglucagon and PC1 levels were normalized to eukaryotic 18S rRNA.

### Statistical Analysis

Log-Rank (Mantel-Cox) Test was used for survival analysis and Graph Pad Prism 5 software was used to generate all the lifespan graphs, as previously reported [20]. The two-tailed student’s t-test was used to compare expression level between the control and the treated cells. All values were reported as mean ± SD and statistical significance defined as *P* <0.05.

## RESULTS

### The lifespan of wild-type C. elegans was shortened by high glucose concentrations

We tested the effect of high levels of glucose on the lifespan of *C. elegans*. Wild- type N2 worms were exposed to either 16 mM or 25 mM glucose, which has been shown to produce glucose toxicity in previous studies in *C. elegans*[1]. As shown in Fig. 1A, the median lifespan of worms exposed to 16 mM glucose was 16 days, with a maximum lifespan of 25 days, compared to a 17-day median lifespan and 27-day maximum lifespan under conditions without high glucose exposure (*P*<0.002). Furthermore, the higher concentration of glucose (25 mM) showed an even stronger adverse effect on lifespan, where the median lifespan of worms exposed to 25 mM glucose was 15 days and the maximum lifespan was 23 days, which was also significantly shorter than the median and maximum of the concurrent no glucose control condition of 20 days and 30 days, respectively (Fig. 1B, *P*<0.0001). These results confirm that high glucose significantly reduce lifespan of wild-type *C. elegans* worms, consistent with results from previous studies [1, 21, 22].

**Figure 2.**
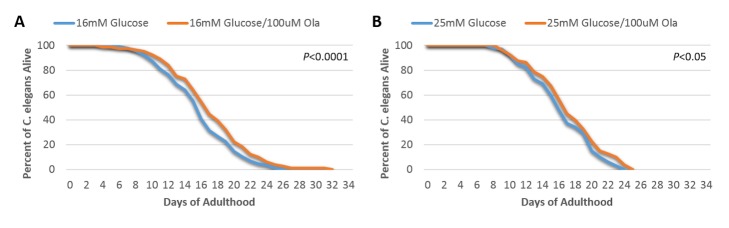
The lifespan-shortening effects of high glucose reduced by Olaparib treatment (**A**) Determination of lifespan for N2 worms exposed to 16 mM glucose together with 100 uM or 0 µM Olaparib (*P*<0.0001, log-rank test, n=100); (**B**) Determination of lifespan for N2 worms exposed to 25 mM glucose together with 100 uM or 0 µM Olaparib (*P*<0.05, log-rank test, n=100).

### Treatment with Olaparib improves shortened lifespan in hyperglycemic conditions in wild-type, but not PARP1 homologue (pme-1) knockout, C. elegans

**Figure 3.**
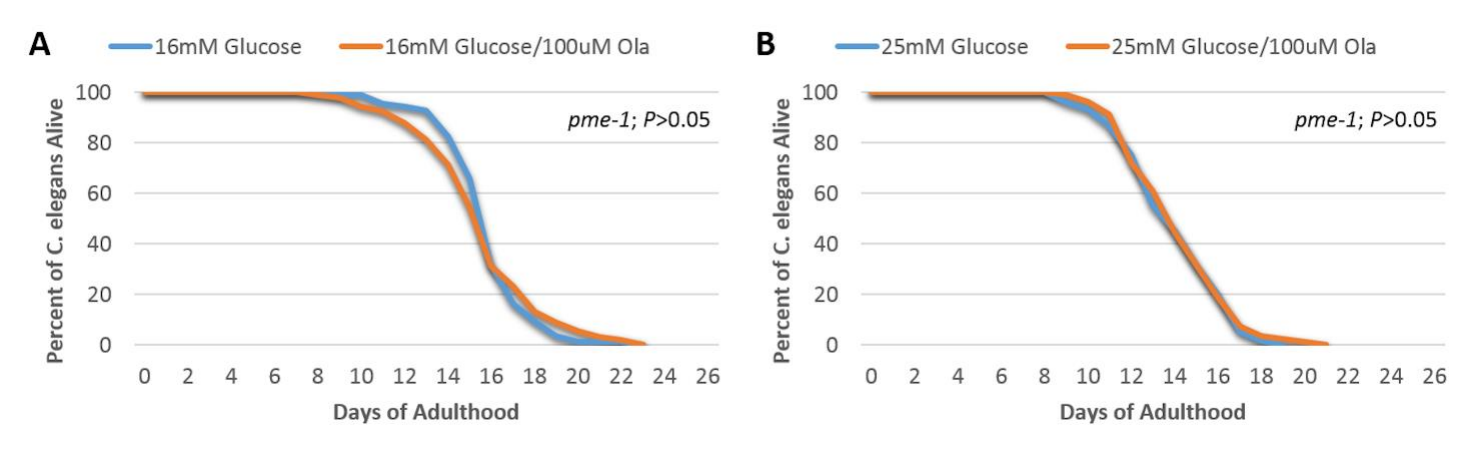
The beneficial effect of Olaparib treatment on *C. elegans* lifespan in the setting of high glucose was target-specific and dependent on the PARP-1 related signaling pathway (**A**) Determination of lifespan for *PARP-1* homolog, *pme-1*, worms exposed to 16 mM glucose together with 100 µM or without Olaparib (*P*>0.05, log-rank test, n=100); (**B**) Determination of lifespan for *pme-1* worms exposed to 25 mM glucose together with 100 µM or 0 µM Olaparib (*P*>0.05, log-rank test, n=100).

We observed that the lifespan of wild-type N2 *C. elegans* was significantly extended by the addition of 100 uM Olaparib in 16 mM glucose (Fig. 2A, *P*<0.0001). We also observed a benefit of 100uM Olarparib in 25 mM glucose (Fig. 2B, *P*<0.05). These results indicate a beneficial effect of PARP-1 inhibition by Olaparib on lifespan under conditions of glucose toxicity. However, upon repeating these experiments in the PARP-1 knock-out mutant strain, *pme-1*, (mutation shown in Supplementary Fig. 2), Olaparib yielded no incremental benefit on lifespan in either 16 mM (Fig. 3A) or 25 mM (Fig. 3B) glucose. These data suggest that the observed beneficial effect of Olaparib on rescuing hyperglycemia-induced short lifespan may be target-specific and directly depends on the PARP-1 signaling pathway.

### TCF7L2 (pop-1) is required for the beneficial effect of Olaparib on lifespan in C. elegans

**Figure 4.**
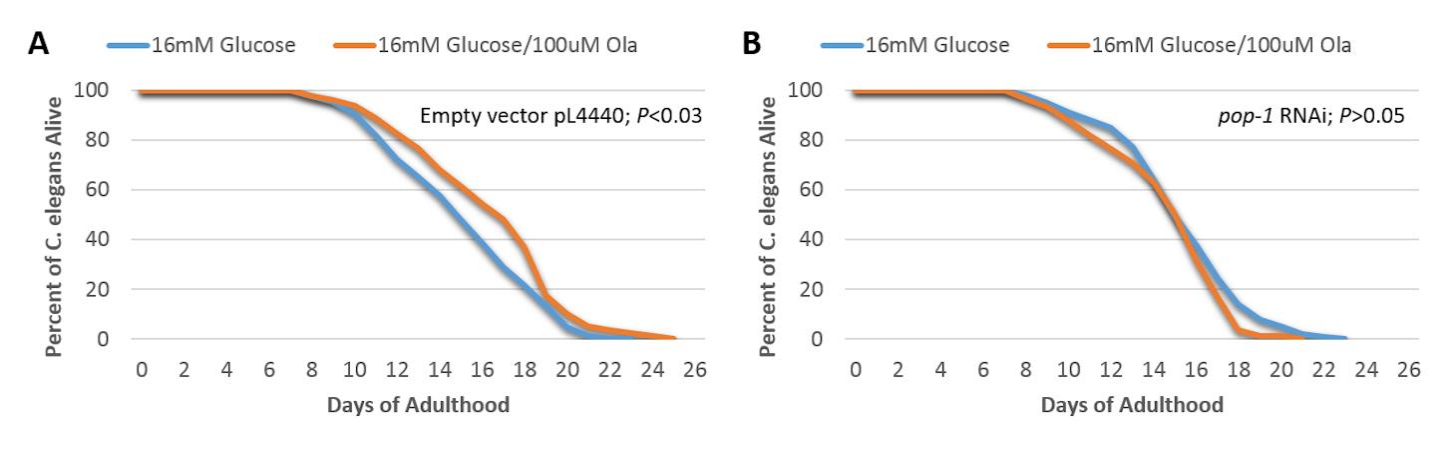
*TCF7L2* homolog, *pop-1*, is required for the beneficial effect in the setting of high glucose of Olaparib treatment on lifespan (**A**) Determination of lifespan for N2 worms fed with HT115 feeding strain containing the pL4440 empty vector and exposed to 16 mM glucose with 100 µM or 0 uM Olaparib (*P*<0.03, log-rank test, n=100); (**B**) Determination of lifespan for N2 worms fed with HT115 feeding strain containing the pL4440 pL4440/*pop-1* RNAi and exposed to 16 mM glucose with 100uM or 0 µM Olaparib (*P*>0.05, log-rank test, n=100).

We investigated whether the effect of PARP-1 inhibition on *C. elegans* lifespan could be mediated by TCF7L2. To examine this possibility, we further compared the lifespan of knock-down worms for the *TCF7L2* homolog, *pop-1* (Supplementary Fig. 3), under hyperglycemic conditions with or without Olaparib. Both the *pop-1* RNAi worms and wild-type N2 control worms were exposed to 16 mM glucose with or without 100 uM Olaparib. As expected, treatment with Olaparib significantly prolonged lifespan of the worms in the wild-type worms exposed to 16 mM glucose (Fig. 4A, *P*<0.03). In contrast, treatment with Olaparib did not have a significant effect on lifespan of the worms in the *pop-1* RNAi group (Fig. 4B). These results suggest that *pop-1 (TCF7L2)* is required for the regulation of *C. elegans* lifespan by a PARP-1 inhibitor in the presence of high glucose.

**Figure 5.**
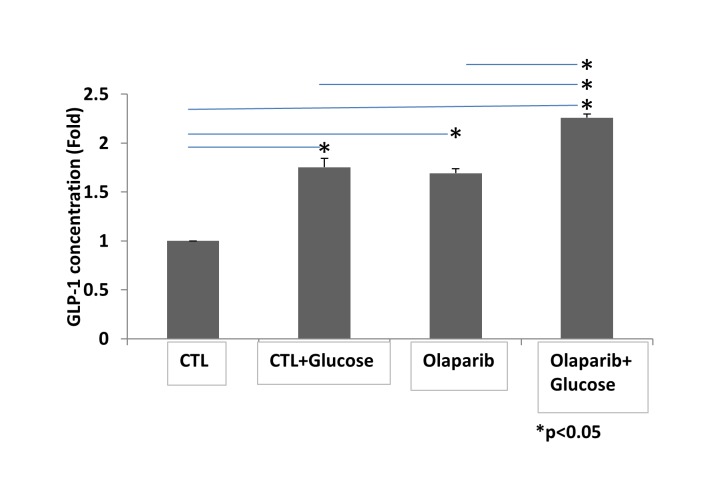
Olaparib enhances promotes GLP-1 secretion in NCI-H716 cells Cells were stimulated for 30 minutes with or without 16 mM glucose. GLP-1 was measured by ELISA. Bars represent the mean of three independent experiments normalized to the control. Error bars indicate standard deviation. Statistical analyses were performed by two-tailed Student’s t-test and significance is denoted by asterisks where *P<0.05.

### Olaparib treatment increases GLP-1 secretion in human NCI-H716 enteroendocrine intestinal cells

Insulin signaling pathways have been implicated in aging and longevity in both worms and mammals [23, 24]. Abnormal insulin homeostasis may underlie the glucose-mediated decrease in lifespan we observed in *C. elegans*, and the beneficial effect of PARP-1 inhibition may result from its downstream effects on insulin production and action. To examine the translational potential of using a PARP-1 inhibitor to treat the sequelae of hyperglycemia in humans, we used the NCI-H716 enteroendocrine cell line that is widely used to study human GLP-1-secretion, an important modulator of insulin production [25]. As shown in Fig. 5, stimulation of NCI-H716 cells with glucose increased GLP-1 release into the medium (p<0.05). Interestingly, Olaparib treatment alone also increased GLP-1 secretion significantly as compared with untreated cells (51.1% average increase; *P*<0.05). Most importantly, levels of active GLP-1 were further increased by combining glucose plus Olaparib as compared with either glucose or Olaparib alone (26.0% and 29.4% average increase, respectively; *P*<0.05). The combination of glucose and Olaparib increased GLP-1 up to 2.2 fold (*P*<0.05). These data suggest that Olaparib induces GLP-1 secretion both in the absence of glucose, and to a greater degree, in its presence.

### Olaparib increases enteroendocrine intestinal cell expression of TCF7L2, GCG and PC1

**Figure 6.**
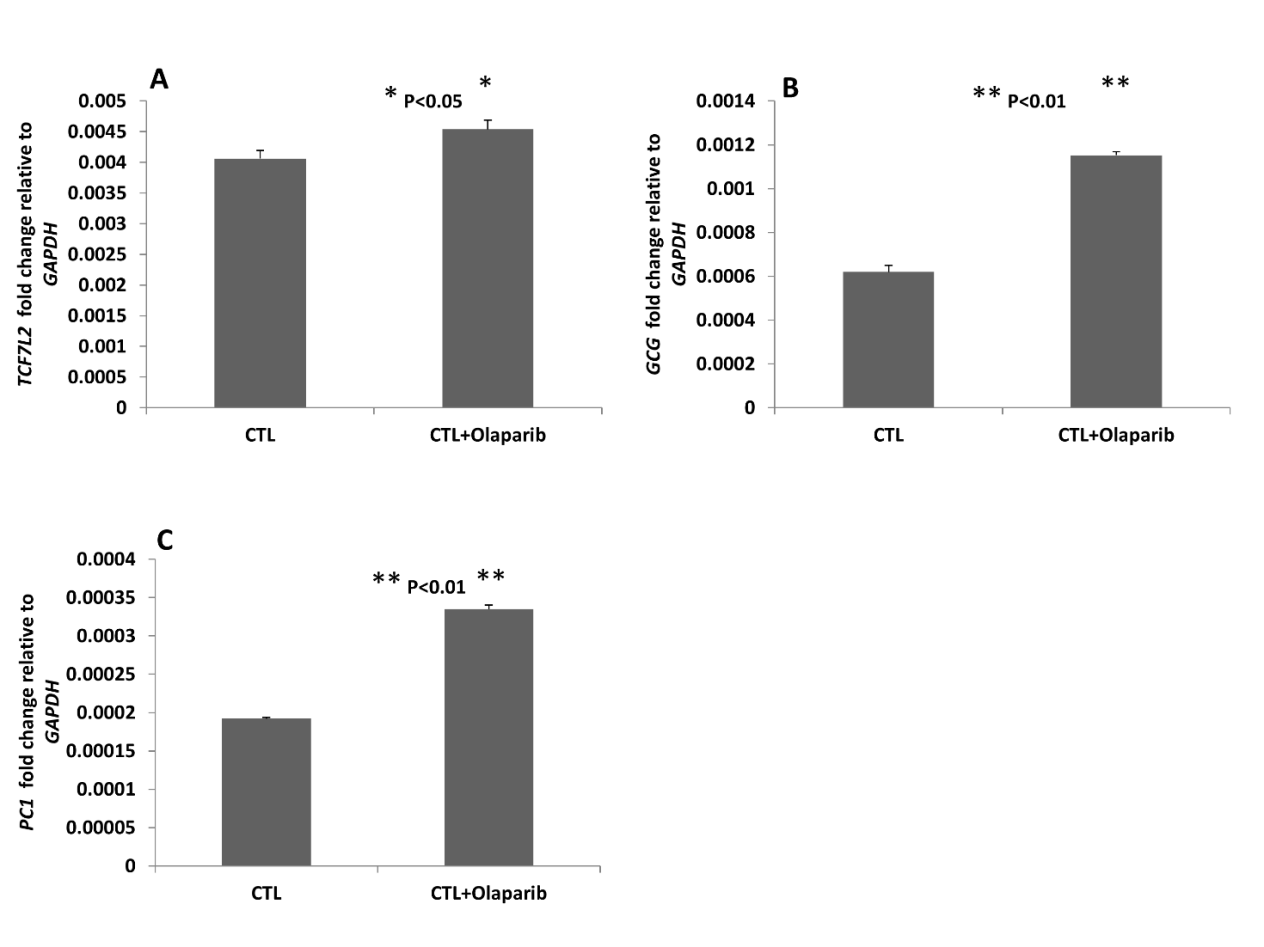
Olaparib treatment of endoendocrine intestinal cells increases expression of (A)*TCF7L2*, (B)*GCG* and (C)*PC1* Relative levels of individual gene expression in the control and the Olaparib treatment groups determined by quantitative PCR with GAPDH normalization. Values are the mean of three independent experiments. Error bars indicate standard deviation. Statistical analyses were performed by two-tailed Student’s t-test and significance is denoted by asterisks where *P<0.05 or **P<0.01.

Given the known interaction between TCF7L2 and the promoter region of the *GCG* gene [9], we hypothesized that PARP-1 interplay with TCF7L2 has an inhibitory effect on *TCF7L2* expression and its downstream stimulation of *GCG* and *PC1* expression. Using quantitative real-time PCR, we compared the expression levels of *TCF7L2* with and without Olaparib treatment for 48 hours in NCI-H716 cells. Our results showed that Olaparib significantly increased *TCF7L2* expression (*P*<0.05) (Fig. 6A). Since we observed that GLP-1 secretion is upregulated by Olaparib treatment, we postulated that both *GCG* and *PC1* expression could also be impacted by PARP-1 inhibition. Indeed, quantitative real time-PCR gene expression results demonstrated that *GC*G and *PC1* expression were significantly increased by up to two-fold with Olaparib treatment (Fig. 6B and 6C, *P*<0.01). These findings support our hypothesis by showing that PARP-1 inhibition with Olaparib reverses the inhibitory effects of PARP-1 on TCF7L2, with subsequent increases in *TCF7L2* and downstream *GCG* and *PC1* gene expression, and net positive effects on GLP-1 production.

## DISCUSSION

Our prior discovery that the genomic region harboring the presumed T2D causal variant at the *TCF7L2* locus, namely rs7903146, binds a protein complex that includes PARP-1 prompted us to functionally evaluate whether PARP-1 inhibition may have anti-diabetic effects.

Although previously implicated in the mediation of diabetes microvascular complications[26], PARP-1 has not been an obvious candidate in the direct pathogenesis of T2D, with a known role principally in DNA damage repair through repair of single-strand [27, 28] and double-strand [29] breaks. A disease role for PARP-1 has been largely the domain of oncology studies, with several PARP-1 inhibitors pursued through clinical trials for various cancers. In particular, Olaparib has been tested in patients with *BRCA*-mutated tumors including breast, ovarian and colorectal cancer in phase II clinical trials [30, 31], with well tolerated side-effects [32]. Interestingly, however, evidence exists for a protective effect of PARP-1 inhibition on the pancreatic β cell, albeit via a mechanism that has not yet been fully elucidated. Specifically, pre-treatment with the PARP-1 inhibitors nicotinamide and 3-aminobenzamide have been shown to reverse a streptozocin-induced decrease in proinsulin synthesis in rats [33]. Furthermore, other gene products related to DNA damage repair have been implicated in diabetes, most recently XRCC4 [34].

Many key metabolic signaling pathways, including insulin, have homologues identified in *C. elegans* [35-39]. Given that PARP-1, as a potential drug target, is also conserved between *C. elegans* and humans, we investigated the mechanism by which PARP-1 inhibition could influence the negative effects of hyperglycemia in the *C. elegans* model animal. It is well-established that increased glucose levels shorten *C. elegans* lifespan, with induction of reactive oxygen species formation and subsequent cellular and systemic damage [1, 21, 22]. Our baseline results confirmed that feeding wild-type *C. elegans* with moderately (16 mM) or severely (25 mM) high glucose diets significantly reduced their lifespan. Conversely, reduced glucose metabolism has been shown to extend lifespan [40, 41], with disruption of certain glycolytic genes also extending lifespan in nematodes [42, 43]. We discovered that PARP-1 inhibition rescued the shortened lifespan of *C. elegans* under hyperglycemic stress, where this pharmacological effect was PARP-1 specific since Olaparib treatment had no effect on mutants for the *PARP-1* homolog, *pme-1*. Beneficial effects of Olaparib on lifespan were also ablated by feeding RNAi knock-down of the *TCF7L2* homolog, *pop-1*, suggesting that TCF7L2 is required to gain therapeutic benefit from PARP-1 inhibition in the context of hyperglycemia. The mechanism by which *pop-1* mediates this effect remains unclear, with further work warranted to elucidate the precise role of the *TCF7L2*/pop-1 homolog in rescuing hyperglycemia-induced toxicity by PARP-1 inhibition. However, our results point to a potential utility of the anti-cancer drug class of PARP-1 inhibitors in the therapeutic context of primary metabolic traits.

**Figure 7.**
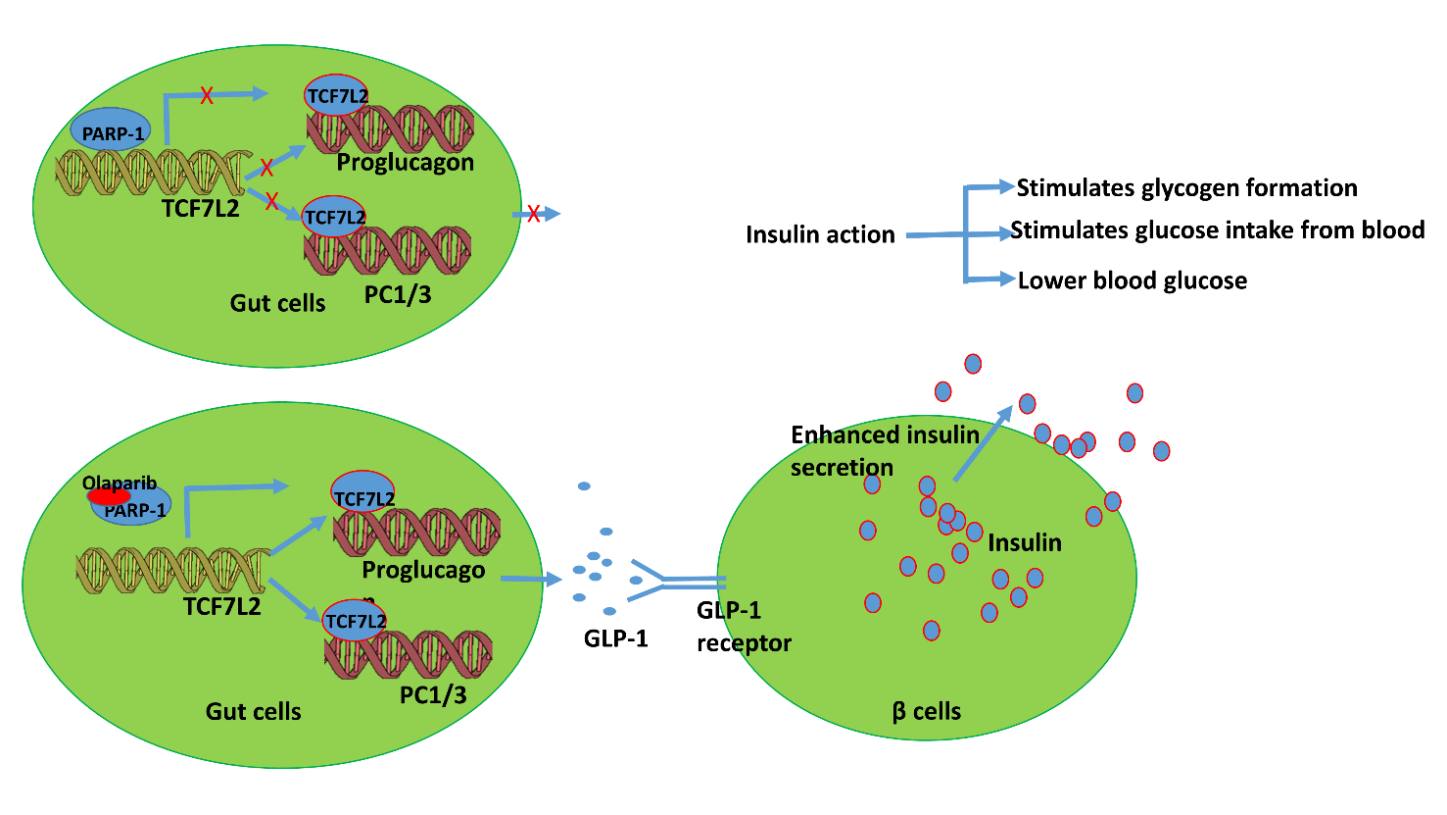
Integrated model to define the role of PARP-1 inhibition in the type 2 diabetes related pathway Olaparib treatment increases the expression of *TCF7L2*, *GCG* and *PC1*, thereby enhancing GLP-1 secretion in the endoendocrine L type gastrointestinal cells and subsequently promoting insulin secretion in the pancreatic β-cell.

Given these results, we used a human cellular model relevant to diabetes mellitus to investigate the mechanistic relationship between TCF7L2 and GLP-1. GLP-1, which plays a crucial role in insulin secretion, is produced in intestinal cells from selective cleavage of the transcription product encoded by *GCG*. Specifically, higher levels of prohormone convertase 1 (encoded by *PC1*) in L cells contribute to the processing of proglucagon to liberate GLP-1 [44]. Indeed, a great deal of effort has been made to improve GLP-1 action for the development of new therapeutic approaches to treat T2D [45, 46]. Since it is known that expression of *GCG* is driven by TCF7L2 in intestinal endocrine L-cells [9, 47] and the NCI-H716 cell line offers a unique model to study the cellular mechanism of GLP-1 secretion in humans [25], we tested the efficacy of a PARP-1 inhibition strategy under hyperglycemic conditions in this cellular model. Collectively, our data suggest an integrated model to support the role of PARP-1 inhibition in T2D-related cellular pathophysiology (Fig. 7). We propose that Olaparib treatment increases intestinal L cell expression of key genes *TCF7L2*, *GCG* and *PC1*, that led to enhanced GLP-1 release in to the gastrointestinal track, consequently augmenting glucose-stimulated insulin secretion in the pancreatic β-cell.

We acknowledge that some debate has surrounded the issue of what protein factors actually bind across the key SNP harbored within *TCF7L2*. One study suggested HMGB1 [48], while another implicated FOXA2 [49]. Although there is a possibility that all these factors play a role in transcriptional control at this genomic region, our current functional data presented here adds further support to our prior findings that PARP-1 is a physiologically important binding partner of TCF7L2 [12]. In addition to support for this factor coming from the rheumatoid arthritis risk variant CCR6DNP regulating *CCR6* via PARP-1[13], it is interesting to note that a recent age at menopause GWAS linked the loci found in that effort to DNA damage response genes[50].

In addition to PARP-1, we recognize that Olaparib can also influence the expression of PARP-2. However, given that we see an influence of Olaparib on lifespan and that independently knocking out *pme-1* (the homolog of *PARP-1* specifically) has a comparable impact, one must conclude that PARP-1 is playing a role in this observation; however, one cannot rule an influence by or on PARP-2 to some degree in this current dataset.

It should be noted that we previously reported an additional gene at the locus, namely *ACSL5*, together with providing further support for *TCF7L2* [51]. There is now an abundance of studies lending a high degree of support for TCF7L2 itself playing a role in T2D pathogenesis at this locus in various tissue contexts. Combined with multiple lines of evidence that the TCF7L2 protein product has a physical interplay with PARP-1, we were motivated us to investigate this relationship further. On the other hand, the role of ACSL5 in the context of type 2 diabetes pathogenesis still needs to be fully elucidated, including how it relates to PARP-1, before one can meaningfully embark on a similar study for that particular factor.

Taken together, these observations suggest that PARP-1 inhibition increases TCF7L2-mediated GLP-1 production. Therefore, pharmacologic PARP-1 inhibition may represent an avenue for therapeutic intervention for T2D. The classical dipeptidyl peptidase-4 (DPP-IV) inhibitors that protect the GLP-1 peptide from degradation have been used successfully as therapeutic reagents for T2D [52]. Our studies suggest a novel mechanism by which the PARP-1 inhibitor, Olaparib, effectively enhances GLP-1 activity in the setting of hyperglycemia and could represent an alternative therapeutic avenue to that of DPP-IV inhibition for T2D.

## 

Supplemental Figure 1 Biological replicates of lifespan assays A) Biological replicate of Figure 1A: High glucose conditions shorten *C. elegans* lifespan. B) Biological replicate of Figure 2A: The lifespan-shortening effects of high glucose reduced by Olaparib treatment (16mM Glucose). C) Biological replicate of Figure 2B: The lifespan-shortening effects of high glucose reduced by Olaparib treatment (25mM Glucose). D) Biological replicate of Figure 3A: The beneficial effect of Olaparib treatment on C. elegans lifespan in the setting of high glucose was target-specific and dependent on the PARP-1 related signaling pathway (16mM Glucose). E) Biological replicate of Figure 4A: *TCF7L2* homolog, *pop-1*, is required for the beneficial effect in the setting of high glucose of Olaparib treatment on lifespan (Empty vector). F) Biological replicate of Figure 4B: *TCF7L2* homolog, *pop-1*, is required for the beneficial effect in the setting of high glucose of Olaparib treatment on lifespan (pop-1 RNAi).

Supplemental Figure 2 Protein sequence alignment of worm pme-1 and human PARP-1. The identical residues are denoted by *.

Supplemental Figure 3 Protein sequence alignment of the DNA-binding HMG box of worm pop-1 and human TCF7L2. The identical residues are denoted by *.

## References

[1] Lee SJ, Murphy CT, Kenyon C (2009). Glucose shortens the life span of C. elegans by downregulating DAF-16/FOXO activity and aquaporin gene expression. Cell Metab, 10:379-391. 19883616 10.1016/j.cmet.2009.10.003 PMC2887095

[2] Finch CE, Ruvkun G (2001). The genetics of aging. Annu Rev Genomics Hum Genet, 2:435-462. 11701657 10.1146/annurev.genom.2.1.435

[3] Visscher PM, Brown MA, McCarthy MI, Yang J (2012). Five years of GWAS discovery. Am J Hum Genet, 90:7-24. 22243964 10.1016/j.ajhg.2011.11.029 PMC3257326

[4] Grant SF, Thorleifsson G, Reynisdottir I, Benediktsson R, Manolescu A, Sainz J, et al (2006). Variant of transcription factor 7-like 2 (TCF7L2) gene confers risk of type 2 diabetes. Nat Genet, 38:320-323. 16415884 10.1038/ng1732

[5] DIAbetes Genetics Replication And Meta-analysis (DIAGRAM) Consortium (2014). Genome-wide trans-ancestry meta-analysis provides insight into the genetic architecture of type 2 diabetes susceptibility. Nat Genet, 46:234-244. 24509480 10.1038/ng.2897 PMC3969612

[6] Wellcome Trust Case Control C, Maller JB, McVean G, Byrnes J, Vukcevic D, Palin K, et al (2012). Bayesian refinement of association signals for 14 loci in 3 common diseases. Nat Genet, 44:1294-1301. 23104008 10.1038/ng.2435 PMC3791416

[7] MacDonald BT, Tamai K, He X (2009). Wnt/beta-catenin signaling: components, mechanisms, and diseases. Dev Cell, 17:9-26. 19619488 10.1016/j.devcel.2009.06.016 PMC2861485

[8] Pearson ER (2009). Translating TCF7L2: from gene to function. Diabetologia, 52:1227-1230. 19387612 10.1007/s00125-009-1356-1

[9] Ni Z, Anini Y, Fang X, Mills G, Brubaker PL, Jin T (2003). Transcriptional activation of the proglucagon gene by lithium and beta-catenin in intestinal endocrine L cells. J Biol Chem, 278:1380-1387. 12421827 10.1074/jbc.M206006200

[10] Langley AK, Suffoletta TJ, Jennings HR (2007). Dipeptidyl peptidase IV inhibitors and the incretin system in type 2 diabetes mellitus. Pharmacotherapy, 27:1163-1180. 17655515 10.1592/phco.27.8.1163

[11] Drucker DJ, Nauck MA (2006). The incretin system: glucagon-like peptide-1 receptor agonists and dipeptidyl peptidase-4 inhibitors in type 2 diabetes. Lancet, 368:1696-1705. 17098089 10.1016/S0140-6736(06)69705-5

[12] Xia Q, Deliard S, Yuan CX, Johnson ME, Grant SF (2015). Characterization of the transcriptional machinery bound across the widely presumed type 2 diabetes causal variant, rs7903146, within TCF7L2. Eur J Hum Genet, 23:103-109. 24667787 10.1038/ejhg.2014.48 PMC4266734

[13] Li G, Cunin P, Wu D, Diogo D, Yang Y, Okada Y, et al (2016). The Rheumatoid Arthritis Risk Variant CCR6DNP Regulates CCR6 via PARP-1. PLoS Genet, 12:e1006292. 27626929 10.1371/journal.pgen.1006292 PMC5023119

[14] Krishnakumar R, Kraus WL (2010). PARP-1 regulates chromatin structure and transcription through a KDM5B-dependent pathway. Mol Cell, 39:736-749. 20832725 10.1016/j.molcel.2010.08.014 PMC2939044

[15] Gong L, Liu FQ, Wang Y, Hou XG, Zhang W, Qin WD, et al (2012). Poly (ADP-ribose) transferase/polymerase-1-deficient mice resistant to age-dependent decrease in beta-cell proliferation. Mol Med, 18:816-824. 22481269 10.2119/molmed.2011.00458 PMC3409279

[16] Idogawa M, Yamada T, Honda K, Sato S, Imai K, Hirohashi S (2005). Poly(ADP-ribose) polymerase-1 is a component of the oncogenic T-cell factor-4/beta-catenin complex. Gastroenterology, 128:1919-1936. 15940627 10.1053/j.gastro.2005.03.007

[17] Idogawa M, Masutani M, Shitashige M, Honda K, Tokino T, Shinomura Y, et al (2007). Ku70 and poly(ADP-ribose) polymerase-1 competitively regulate beta-catenin and T-cell factor-4-mediated gene transactivation: possible linkage of DNA damage recognition and Wnt signaling. Cancer Res, 67:911-918. 17283121 10.1158/0008-5472.CAN-06-2360

[18] Pieper AA, Brat DJ, Krug DK, Watkins CC, Gupta A, Blackshaw S, et al (1999). Poly(ADP-ribose) polymerase-deficient mice are protected from streptozotocin-induced diabetes. Proc Natl Acad Sci U S A, 96:3059-3064. 10077636 10.1073/pnas.96.6.3059 PMC15894

[19] Li B, Luo C, Chowdhury S, Gao ZH, Liu JL (2013). Parp1 deficient mice are protected from streptozotocin-induced diabetes but not caerulein-induced pancreatitis, independent of the induction of Reg family genes. Regul Pept, 186:83-91. 23954400 10.1016/j.regpep.2013.07.005

[20] Dingley S, Polyak E, Lightfoot R, Ostrovsky J, Rao M, Greco T, et al (2010). Mitochondrial respiratory chain dysfunction variably increases oxidant stress in Caenorhabditis elegans. Mitochondrion, 10:125-136. 19900588 10.1016/j.mito.2009.11.003 PMC3638869

[21] Schulz TJ, Zarse K, Voigt A, Urban N, Birringer M, Ristow M (2007). Glucose restriction extends Caenorhabditis elegans life span by inducing mitochondrial respiration and increasing oxidative stress. Cell Metab, 6:280-293. 17908557 10.1016/j.cmet.2007.08.011

[22] Schlotterer A, Kukudov G, Bozorgmehr F, Hutter H, Du X, Oikonomou D, et al (2009). C. elegans as model for the study of high glucose- mediated life span reduction. Diabetes, 58:2450-2456. 19675139 10.2337/db09-0567 PMC2768179

[23] Kenyon CJ (2010). The genetics of ageing. Nature, 464:504-512. 20336132 10.1038/nature08980

[24] van Heemst D, Beekman M, Mooijaart SP, Heijmans BT, Brandt BW, Zwaan BJ, et al (2005). Reduced insulin/IGF-1 signalling and human longevity. Aging Cell, 4:79-85. 15771611 10.1111/j.1474-9728.2005.00148.x

[25] Reimer RA, Darimont C, Gremlich S, Nicolas-Metral V, Ruegg UT, Mace K (2001). A human cellular model for studying the regulation of glucagon-like peptide-1 secretion. Endocrinology, 142:4522-4528. 11564718 10.1210/endo.142.10.8415

[26] Du X, Matsumura T, Edelstein D, Rossetti L, Zsengeller Z, Szabo C, et al (2003). Inhibition of GAPDH activity by poly(ADP-ribose) polymerase activates three major pathways of hyperglycemic damage in endothelial cells. J Clin Invest, 112:1049-1057. 14523042 10.1172/JCI18127 PMC198524

[27] Caldecott KW (2007). Mammalian single-strand break repair: mechanisms and links with chromatin. DNA Repair (Amst), 6:443-453. 17118715 10.1016/j.dnarep.2006.10.006

[28] Woodhouse BC, Dianov GL (2008). Poly ADP-ribose polymerase-1: an international molecule of mystery. DNA Repair (Amst), 7:1077-1086. 18468963 10.1016/j.dnarep.2008.03.009

[29] Rouleau M, Patel A, Hendzel MJ, Kaufmann SH, Poirier GG (2010). PARP inhibition: PARP1 and beyond. Nat Rev Cancer, 10:293-301. 20200537 10.1038/nrc2812 PMC2910902

[30] Marchetti C, Imperiale L, Gasparri ML, Palaia I, Pignata S, Boni T, et al (2012). Olaparib, PARP1 inhibitor in ovarian cancer. Expert Opin Investig Drugs, 21:1575-1584. 10.1517/13543784.2012.707189 22788971

[31] Glendenning J, Tutt A (2011). PARP inhibitors--current status and the walk towards early breast cancer. Breast, 20 Suppl 3:S12-19. 10.1016/S0960-9776(11)70288-0 22015278

[32] Ledermann J, Harter P, Gourley C, Friedlander M, Vergote I, Rustin G, et al (2012). Olaparib maintenance therapy in platinum-sensitive relapsed ovarian cancer. N Engl J Med, 366:1382-1392. 22452356 10.1056/NEJMoa1105535

[33] Uchigata Y, Yamamoto H, Nagai H, Okamoto H (1983). Effect of poly(ADP-ribose) synthetase inhibitor administration to rats before and after injection of alloxan and streptozotocin on islet proinsulin synthesis. Diabetes, 32:316-318. 6299867 10.2337/diab.32.4.316

[34] Dooley J, Tian L, Schonefeldt S, Delghingaro-Augusto V, Garcia-Perez JE, Pasciuto E, et al (2016). Genetic predisposition for beta cell fragility underlies type 1 and type 2 diabetes. Nat Genet, 48:519-527. 26998692 10.1038/ng.3531 PMC5584070

[35] Consortium CeS (1998). Genome sequence of the nematode C. elegans: a platform for investigating biology. Science, 282:2012-2018. 9851916 10.1126/science.282.5396.2012

[36] Sonnhammer EL, Durbin R (1997). Analysis of protein domain families in Caenorhabditis elegans. Genomics, 46:200-216. 9417907 10.1006/geno.1997.4989

[37] Lai CH, Chou CY, Ch’ang LY, Liu CS, Lin W (2000). Identification of novel human genes evolutionarily conserved in Caenorhabditis elegans by comparative proteomics. Genome Res, 10:703-713. 10810093 10.1101/gr.10.5.703 PMC310876

[38] Kuwabara PE, O’Neil N (2001). The use of functional genomics in C. elegans for studying human development and disease. J Inherit Metab Dis, 24:127-138. 11405335 10.1023/a:1010306731764

[39] Harris TW, Chen N, Cunningham F, Tello-Ruiz M, Antoshechkin I, Bastiani C, et al (2004). WormBase: a multi-species resource for nematode biology and genomics. Nucleic Acids Res, 32:D411-417. 14681445 10.1093/nar/gkh066 PMC308800

[40] Yokoyama K, Fukumoto K, Murakami T, Harada S, Hosono R, Wadhwa R, et al (2002). Extended longevity of Caenorhabditis elegans by knocking in extra copies of hsp70F, a homolog of mot-2 (mortalin)/mthsp70/Grp75. FEBS Lett, 516:53-57. 11959102 10.1016/s0014-5793(02)02470-5

[41] Urban N, Tsitsipatis D, Gille A, Hamann I, Hou X, Klotz LO (2014). Modulation of cellular thiol status affects FoxO activity and life span. Free Radic Biol Med, 75 Suppl 1:S53. 10.1016/j.freeradbiomed.2014.10.826 26461409

[42] Hamilton B, Dong Y, Shindo M, Liu W, Odell I, Ruvkun G, et al (2005). A systematic RNAi screen for longevity genes in C. elegans. Genes Dev, 19:1544-1555. 15998808 10.1101/gad.1308205 PMC1172061

[43] Lee SS, Lee RY, Fraser AG, Kamath RS, Ahringer J, Ruvkun G (2003). A systematic RNAi screen identifies a critical role for mitochondria in C. elegans longevity. Nat Genet, 33:40-48. 12447374 10.1038/ng1056

[44] Scopsi L, Gullo M, Rilke F, Martin S, Steiner DF (1995). Proprotein convertases (PC1/PC3 and PC2) in normal and neoplastic human tissues: their use as markers of neuroendocrine differentiation. J Clin Endocrinol Metab, 80:294-301. 7829629 10.1210/jcem.80.1.7829629

[45] Donnelly D (2012). The structure and function of the glucagon-like peptide-1 receptor and its ligands. Br J Pharmacol, 166:27-41. 21950636 10.1111/j.1476-5381.2011.01687.x PMC3415635

[46] Ahren B (2011). GLP-1 for type 2 diabetes. Exp Cell Res, 317:1239-1245. 21237153 10.1016/j.yexcr.2011.01.010

[47] Yi F, Brubaker PL, Jin T (2005). TCF-4 mediates cell type-specific regulation of proglucagon gene expression by beta-catenin and glycogen synthase kinase-3beta. J Biol Chem, 280:1457-1464. 15525634 10.1074/jbc.M411487200

[48] Zhou Y, Oskolkov N, Shcherbina L, Ratti J, Kock KH, Su J, et al (2016). HMGB1 binds to the rs7903146 locus in TCF7L2 in human pancreatic islets. Mol Cell Endocrinol, 430:138-145. 26845344 10.1016/j.mce.2016.01.027

[49] Gaulton KJ, Ferreira T, Lee Y, Raimondo A, Magi R, Reschen ME, et al (2015). Genetic fine mapping and genomic annotation defines causal mechanisms at type 2 diabetes susceptibility loci. Nat Genet, 47:1415-1425. 26551672 10.1038/ng.3437 PMC4666734

[50] Day FR, Ruth KS, Thompson DJ, Lunetta KL, Pervjakova N, Chasman DI, et al (2015). Large-scale genomic analyses link reproductive aging to hypothalamic signaling, breast cancer susceptibility and BRCA1-mediated DNA repair. Nat Genet, 47:1294-1303. 26414677 10.1038/ng.3412 PMC4661791

[51] Xia Q, Chesi A, Manduchi E, Johnston BT, Lu S, Leonard ME, et al (2016). The type 2 diabetes presumed causal variant within TCF7L2 resides in an element that controls the expression of ACSL5. Diabetologia. 10.1007/s00125-016-4077-2 27539148

[52] Tuduri E, Lopez M, Dieguez C, Nadal A, Nogueiras R (2016). Glucagon-Like Peptide 1 Analogs and their Effects on Pancreatic Islets. Trends Endocrinol Metab, 27:304-318. 27062006 10.1016/j.tem.2016.03.004

