# Supplementary figures and images for: PARP-1 Inhibition Rescues Short Lifespan in Hyperglycemic C. Elegans And Improves GLP-1 Secretion in Human Cells
Source: Aging Dis. 2018 Feb 1;9(1):17–30. doi: 10.14336/AD.2017.0230 (PMC5772855; doi:10.14336/AD.2017.0230)

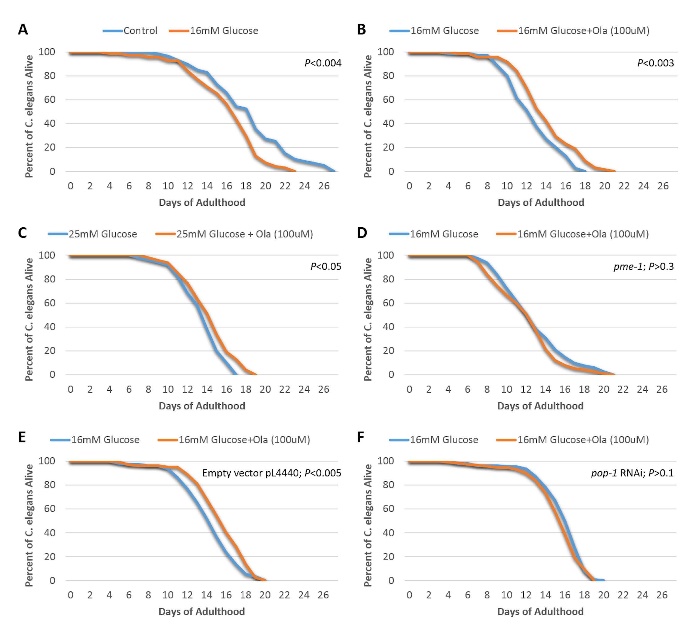

Supplement: Supplemental Figure 1 — A) Biological replicate of Figure 1A: High glucose conditions shorten C. elegans lifespan. B) Biological replicate of Figure 2A: The lifespan-shortening effects of high glucose reduced by Olaparib treatment (16mM Glucose). C) Biological replicate of Figure 2B: The lifespan-shortening effects of high glucose reduced by Olaparib treatment (25mM Glucose). D) Biological replicate of Figure 3A: The beneficial effect of Olaparib treatment on C. elegans lifespan in the setting of high glucose was target-specific and dependent on the PARP-1 related signaling pathway (16mM Glucose). E) Biological replicate of Figure 4A: TCF7L2 homolog, pop-1, is required for the beneficial effect in the setting of high glucose of Olaparib treatment on lifespan (Empty vector). F) Biological replicate of Figure 4B: TCF7L2 homolog, pop-1, is required for the beneficial effect in the setting of high glucose of Olaparib treatment on lifespan (pop-1 RNAi). [file ad-9-1-17-g8.jpg]

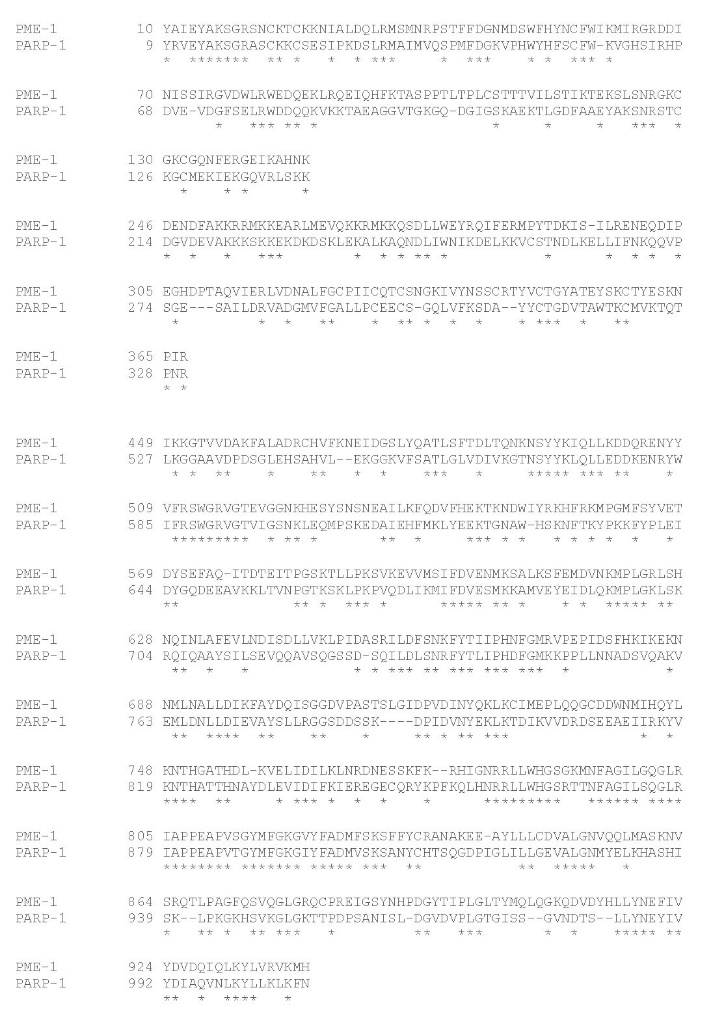

Supplement: Supplemental Figure 2 — Protein sequence alignment of worm pme-1 and human PARP-1. The identical residues are denoted by *. [file ad-9-1-17-g9.jpg]

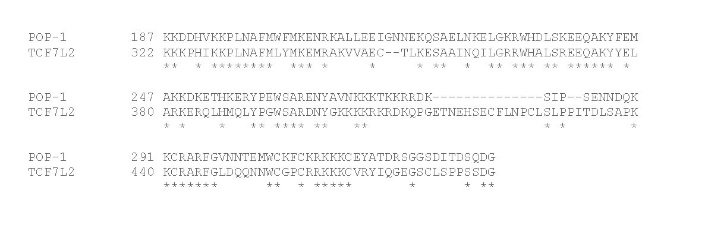

Supplement: Supplemental Figure 3 — Protein sequence alignment of the DNA-binding HMG box of worm pop-1 and human TCF7L2. The identical residues are denoted by *. [file ad-9-1-17-g10.jpg]
